# A greener and simpler lutein extraction process applied to heterotrophically-grown *Scenedesmus almeriensis*

**DOI:** 10.1007/s00449-026-03319-5

**Published:** 2026-03-26

**Authors:** Cristobal Camarena-Bernard, Diego Lopez Salas, Théo Jullien, Rafik Balti, Victor Pozzobon

**Affiliations:** 1 Laboratoire de Génie des Procédés et Matériaux, Centre Européen de Biotechnologie et de Bioéconomie (CEBB), Université Paris-Saclay, CentraleSupélec, 3 rue des Rouges Terres, 51110 Pomacle, France; 2 Instituto de Estudios Superiores de Occidente (ITESO), 45604 Tlaquepaque, Jalisco Mexico

**Keywords:** Microalgae, Scenedesmus almeriensis, Lutein ; Sustainable extraction, Pigment stability, Cell-wall strength

## Abstract

**Abstract:**

In this study, the extraction of lutein from the microalgae *Scenedesmus almeriensis* cultivated under heterotrophic conditions was investigated with the aim of making the process more environmentally friendly, less toxic for subsequent food and feed usage, and with a reduced ecological footprint. The results show that freeze-drying the biomass does not increase lutein recovery (p > 0.05). Additionally, $4^{\circ }$C storage of wet biomass is a viable alternative (15 % loss) to -$20^{\circ }$C storage. Ethanolic KOH solution was found to be more effective (+32.8 %) for lutein extraction compared to aqueous solution, although saponification ultimately did not provide benefits for lutein extraction. Among the solvents compared, ethanol (0.85 ± 0.01 mg $\hbox {gDW}^{-1}$) performed as well as acetone (0.91 ± 0.11 mg $\hbox {gDW}^{-1}$) or isopropanol (0.87 ± 0.01 mg $\hbox {gDW}^{-1}$) and was better than methanol (0.61 ± 0.01 mg $\hbox {gDW}^{-1}$) or 2-methyltetrahydrofuran (0.38 ± 0.08 mg $\hbox {gDW}^{-1}$). In addition, a single bead-beating round allows for the recovery of $99^+$ % of the pigments, and the maceration step appears facultative. Ethanolic extracts remained stable at both $4^{\circ }$C and –$20^{\circ }$C for at least one month. All these findings were agglomerated and proposed as a streamlined protocol. Finally, it was observed that cells of *S. almeriensis* grown under heterotrophic conditions offer less disruption resistance than their autotrophic counterparts.

**Graphic abstract:**

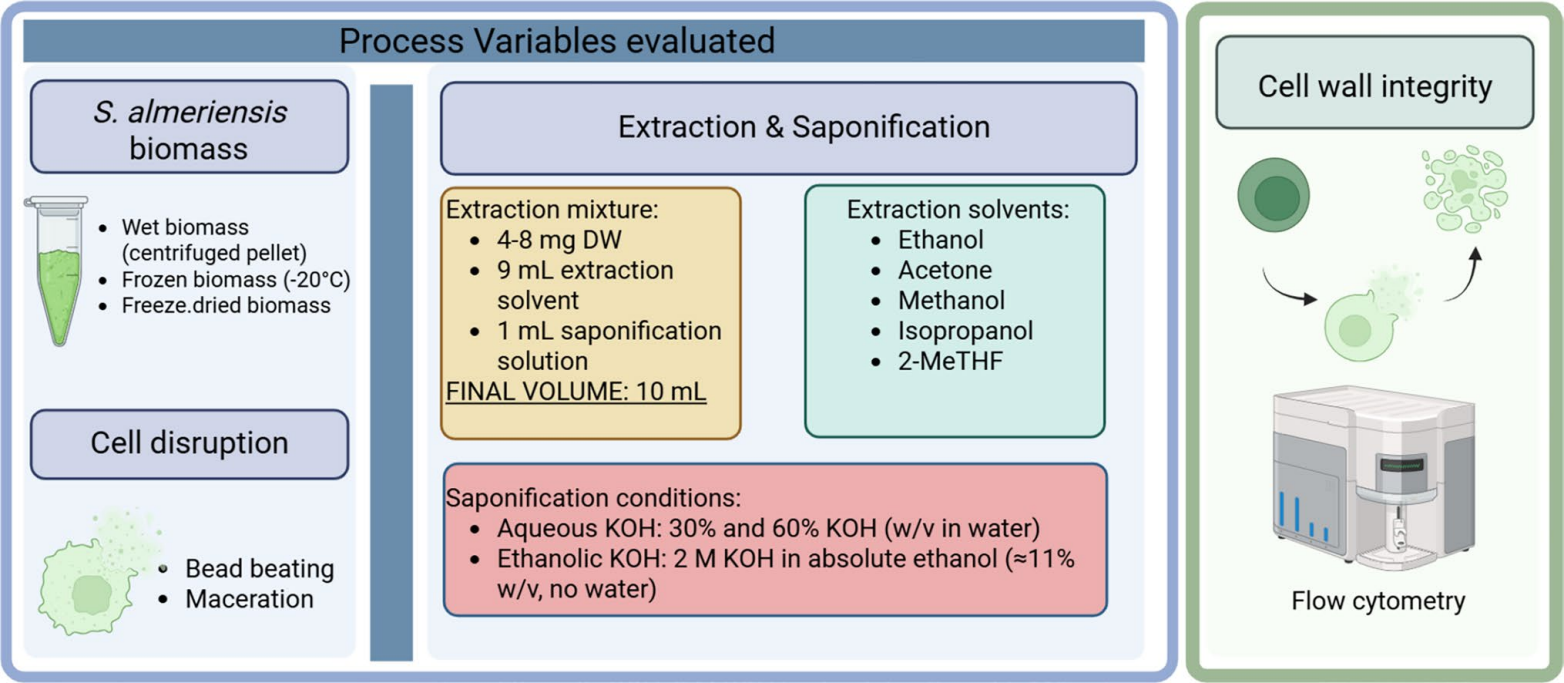

## Introduction

Lutein, a carotenoid found in various foods such as green leafy vegetables, carrots, and eggs, has attracted considerable interest due to its numerous health benefits for humans [41]. Among its functions, its antioxidant capacity and its role in protecting ocular health, particularly in preventing diseases such as age-related macular degeneration, stand out [50]. In addition, its capability to cross the blood-brain barrier positions it as a supplement for cognitive reinforcement and neurodegenerative conditions prevention [27]. Currently, commercial production of lutein primarily relies on marigold flowers. However, in light of agroecological considerations, microalgae have emerged as a promising alternative. Despite its potential, the commercial production of lutein from microalgae faces significant challenges, with downstream processing being a prominent bottleneck. Efficient extraction of lutein and other carotenoids from microalgae, such as *Scenedesmus almeriensis*, has become a crucial research area to improve the commercial viability of these compounds. In addition to optimizing lutein production, there is a growing urgency to transition to more sustainable and environmentally friendly extraction processes. Pressure to use solvents accepted for human use and consumption and reduce the environmental footprint of industrial processes has driven the search for more efficient and less polluting alternatives [21].

Since drying constitutes the primary energy and time consumption in the process of carotenoid extraction from biomass [54], it is necessary to optimize the conditions of these factors for wet extraction in order to reduce the impact of this process as much as possible and make it economically feasible [44]. Lutein recovery generally consists of biomass pretreatment and solvent extraction. Additionally, certain studies incorporate saponification and/or thermal treatment steps [13], yet there remains no consensus regarding the necessity of these two steps. The pretreatment is important to break the cells and allow direct contact of the solvent with the lutein, which is located inside the chloroplast. Numerous pretreatments, both mechanical and chemical, have been proposed, such as bead beating homogenization [13], ultrasonication [19, 55], microwave [2, 35], French press [14], ball milling [39], and alkaline treatment [14]. The solvent extraction is performed with organic solvents of different polarity, such as methanol, ethanol, acetone, dichloromethane, diethyl ether, and hexane, but also with supercritical fluids [36], like $\hbox {CO}_2$. A comprehensive analysis has been done by Saini et al. [46].

Carotenoids are C40 isoprenoid compounds characterized by an extended conjugated double-bond system, which strongly influences their physicochemical properties and extraction behavior. They are commonly divided into two main groups: carotenes, which are purely hydrocarbon molecules such as lycopene and the cyclic forms $\alpha$-carotene and $\beta$-carotene, and xanthophylls, which are oxygenated derivatives formed through oxidative modifications. The oxidation of $\alpha$-carotene leads to the formation of lutein, whereas $\beta$-carotene oxidation yields other xanthophylls such as zeaxanthin, violaxanthin, canthaxanthin, and astaxanthin. The presence of hydroxyl, keto, or epoxy functional groups increases the polarity of xanthophylls compared to carotenes, a key feature that influences their solubility, interaction with solvents, and response to treatments such as saponification during extraction.

The treatment of biomass with alkaline solutions during the extraction of lutein has been approached with three objectives. Firstly, as a chemical mechanism of cell disruption by its action on cell membrane lipids, allowing the solvent to penetrate and extract lutein [15, 47]. Secondly, as a saponification process to obtain free lutein, in the case of esterified lutein [12]. Finally, the saponification step serves as a way to remove chlorophyll from the extract, avoiding the spectral overlap with the species found within the carotenoid family [39]. While in plants lutein is generally esterified, such as in marigold flower petals [33], in microalgae it is generally found in free form [26]. However, there is no general consensus, and it appears that various ratios of free/esterified lutein can be found in microalgae depending on the species and culture conditions [23].

The utilization of heat in the extraction procedure is also a topic of discussion. While it is recognized that lutein and other carotenoids are heat-sensitive, with degradation starting at temperatures between $40^{\circ }$C and $60^{\circ }$C, there is a notion that elevated temperatures enhance the efficacy of solvents and, if applicable, saponification, thereby accelerating the reactions [34, 40, 46].

Within the diverse microalgal taxa investigated for carotenoid production, species of the genus *Scenedesmus* have attracted particular attention due to their robust growth, adaptability to a wide range of cultivation conditions, and naturally high lutein content [25]. Members of this genus are fast-growing green microalgae capable of thriving under both autotrophic and heterotrophic modes, making them suitable candidates for large-scale production systems [56]. Their cellular architecture favors the accumulation of xanthophylls such as lutein [8], while their tolerance to environmental stressors enables process optimization through modulation of light intensity, nutrient availability, or carbon source [29]. Consequently, *Scenedesmus* species are increasingly regarded as promising biotechnological platforms for the sustainable and efficient production of lutein and other high-value carotenoids [32].

Among them, *S. almeriensis* is a promising microalga for lutein production. When cultivated under phototrophic conditions, it can produce up to 8.5 mg $\hbox {gDW}^{-1}$ (Dry Weight basis) of lutein , with a biomass productivity of 0.13 g $\hbox {L}^{-1}$ $\hbox {d}^{-1}$, amounting to a lutein productivity of 1.1 mg $\hbox {L}^{-1}$ $\hbox {d}^{-1}$ [38]. Even though in heterotrophy the amount of lutein from *S. almeriensis* is lower than in phototrophy, the higher amount of biomass produced shows an increase in productivity of up to 11.68 mg $\hbox {L}^{-1}$ $\hbox {d}^{-1}$, one of the highest reported productivities to date [9]. For comparison, marigold (*Tagetes erecta*), the current industrial source of lutein, produces approximately 10.6 kg $\hbox {ha}^{-1}$ $\hbox {yr}^{-1}$ of lutein [6]. In contrast, microalgal cultivation of *Scenedesmus*, under open-pond conditions and assuming typical lutein contents, has been estimated to reach significantly higher areal productivities [18, 33], highlighting the strong potential of microalgae as an alternative and sustainable lutein source. In this context, another advantage of the heterotrophic mode of production lies in the production process itself. It allows for a more compact implementation compared to phototrophy. Also, skilled workers and production facilities are available to lead the process owing to its similarity with beer and baker’s yeast productions.

This study pursues multiple objectives centered on the development of a more efficient and sustainable extraction process for lutein and related carotenoids. The first goal is to compare lutein extraction efficiency between dry and wet biomass, an underrepresented trend in the literature. Additionally, the relevance of saponification using either aqueous or ethanolic KOH is evaluated, as the relevance of its use is debated. Five solvents have also been compared to identify the most suitable extraction medium, while also considering environmental impact and effects on human health. As an opening toward generalizability, the study investigates differences in cell wall resistance of *Scenedesmus almeriensis* cultivated under heterotrophic versus phototrophic conditions, to assess variations in susceptibility to cell disruption. A similar analysis is performed using *Chlorella vulgaris* cells grown under both metabolic regimes, offering comparative insights across species.

## Materials and methods

### Microorganism and culture conditions

A strain of *Scenedesmus almeriensis* was generously donated by Prof. Francisco Gabriel Acien of the University of Almeria. The strain has been cultivated under heterotrophic conditions in the laboratory using 4x B3N medium supplemented with 40 g $\hbox {L}^{-1}$ glucose. B3N culture medium contained (per liter): $\hbox {NaNO}_3$ (750 mg), $\hbox {MgSO}_4$ 7$\hbox {H}_2$O (75 mg), NaCl (25 mg), $\hbox {K}_2 \hbox {HPO}_4$ (75 mg), $\hbox {KH}_2 \hbox {PO}_4$ (175 mg), $\hbox {CaCl}_2$ 2$\hbox {H}_2$O (25 mg), $\hbox {ZnSO}_4$ 7$\hbox {H}_2$O (8.82 mg), $\hbox {MnCl}_2$ 4$\hbox {H}_2$O (1.44 mg), $\hbox {MoO}_3$ (0.71 mg) $\hbox {CuSO}_4$ 5$\hbox {H}_2$O (1.57 mg), CO($\hbox {NO}_3$)$_2$ 6$\hbox {H}_2$O (0.49 mg), $\hbox {H}_3 \hbox {BO}_3$ (11.42 mg), EDTA (50.0 mg), KOH (31 mg) and $\hbox {FeSO}_4$ 7$\hbox {H}_2$O (4.98 mg) [4]. The culture was continuously agitated (100 rpm) in a dark incubator at $30^{\circ }$C and subcultured weekly to replace nutrients. Detailed behavior of the culture has been described previously, and the reader is referred to the dedicated article. Still, key aspects can be summarized as follows. *Scenedesmus almeriensis* exhibits a 0.5 $\hbox {g}_{Biomass}$ $\hbox {g}_{Glucose}^{-1}$ yield (final biomass concentration around 18 g $\hbox {L}^{-1}$) associated with a growth rate around 1.2−1.4 1/day, for cultures taking three days to reach the stationary phase (1/100 passaging). Finally, the lutein content is halved compared to phototrophically grown cells [9].

For every trial, the culture was harvested during the exponential phase, and the samples were centrifuged ($4^{\circ }$C, 11,000 rpm, 10 min), the supernatant was discarded, and the pellet was washed with distilled water, divided into 1 mL tubes, and then centrifuged again. For the wet biomass trial, the tubes were divided in three; one part was stored at $4^{\circ }$C, another one at -$20^{\circ }$C ( temperatures chosen based on the availability of conventional equipment) and the last part was freeze-dried (1-day primary drying, 1-day secondary drying, Christ alpha 1-2 LD +) before being stored at -$20^{\circ }$C. All samples were protected from light.

### Biomass conditioning and storage test

The first experiment was designed to evaluate the impact of biomass conditioning and short-term storage on carotenoid extractability, with the aim of assessing whether wet biomass could serve as a viable alternative to freeze-dried material and thereby reduce downstream processing requirements. To do so, an extraction of carotenoids from wet biomass stored, for three days, at $4^{\circ }$C and -$20^{\circ }$C was compared with the extraction obtained from biomass dried by freeze-drying. For this, all samples were resuspended in 1 mL of distilled water, and pigments were extracted using a modified protocol described by Pozzobon and Camarena-Bernard [42]. Cell disruption was performed by mixing 1 mL of laboratory-grade inert sand (Fisher Scientific Code: 10132590) and 9 mL of methanol. Cell disruption was carried out in a high-speed benchtop homogenizer (MP Biomedicals™ FastPrep-24™ 5 G Instrument, Fisherbrand, Waltham, MA, USA) at 6.5 m $\hbox {s}^{-1}$ in two cycles of 30 s with a 60-second pause. The samples were then macerated at room temperature in a rotator (Stuart Rotator SB3) at 10 rpm for 60 min. Finally, the samples were filtered through a 0.22 $\upmu$m pore filter to separate particles before passing them through a high-pressure liquid chromatograph. All this protocol was conducted with light protection around the samples.

### Saponification treatment

The second set of experiments was designed to evaluate the role of saponification in lutein extraction and to assess how the chemical nature of the alkaline medium influences pigment recovery. First, the effect of saponification was compared using aqueous KOH solutions at concentrations of 30 and 60% w/v (as an extreme condition to test the upper limit of the process). For this, the same disruption procedure described above was followed using wet biomass. The biomass was pelleted before being resuspended in 1 mL of aqueous KOH solution (to which 1 mL of sand was added). Then it was presented to the homogenizer. After cell disruption, solvent (acetone instead of methanol) was added to the mixture, and the tubes were allowed to macerate in the rotator for 1 h at room temperature at 10 rpm. Overall, the macerating tube contained disrupted biomass, 9 mL of acetone, and 1 mL of aqueous KOH. The KOH solution was replaced with 1 mL of distilled water for the control group.

Second, since carotenoids like lutein are not soluble in water, the extraction of pigments was compared using a 2 M ethanolic KOH solution (i.e., 11% w/v) instead of an aqueous one. This was performed this way to increase the solvent-to-biomass ratio while reducing the volume of water in the mixture. Additionally, a test replacing acetone with ethanol as an extraction solvent was included. This was done as a control to discard any interaction between acetone and the ethanol in the saponification solution. The rest of the extraction steps remained as stated before. Overall, the macerating tube contained disrupted biomass, 9 mL of acetone or ethanol, and 1 mL of ethanolic KOH.

### Solvent evaluation

This set of experiments was designed to compare the efficiency of different extraction solvents for lutein and other carotenoids. It also aimed at disentangling solvent effects from those related to cell disruption and downstream processing steps. Particular emphasis was placed on identifying solvent systems compatible with ethanol-based workflows, evaluating the necessity of repeated extraction and maceration steps, and assessing the short-term stability of ethanolic extracts under common storage conditions. With this aim, solvent extracting efficiency was assessed for: methanol, acetone, ethanol, isopropanol, and 2-methyltetrahydrofuran (2-MeTHF), the latter supplemented with 50 ppm of butylated hydroxytoluene (BHT) as an antioxidant.

To ensure that solvent performance was not influenced by the cell disruption step, the biomass was first disrupted in water using an ultrasonic probe (VC 50 1/4" Microtip, Vibra-Cell Sonics VCX130, Newtown, CT, USA), and solvents were added afterward. This method had previously been optimized and showed comparable carotenoid recovery yields to those obtained with high-speed benchtop homogenizers. It voids a direct comparison between the sets of experiments carried out with different homogenization methods. The sonication protocol consisted of two 60-second pulses at 130?W and 20?kHz, each followed by a 60-second pause. Post-disruption, 9?mL of solvent and 1?mL of 2?M ethanolic KOH were added for saponification.

In a separate assay to determine the efficiency of pigment extraction by bead beating in ethanol, a repeated extraction test was conducted on the same biomass sample. *S. almeriensis* cells were washed twice, and the supernatant was discarded. The disruption was carried out following the method described in section *2.2. Biomass conditioning and storage test*, substituting methanol for ethanol. The extraction involved three sequential rounds. After the first disruption, 60% of the extract was filtered and labeled as the first extract. An equal volume of ethanol was added to the remaining biomass for the second round, and the process was repeated a third time. Pigments were quantified in each extract, and the amount recovered at each step was calculated by correcting the concentration for the remaining pigments from the prior extraction. All extractions were performed in triplicate.

Once bead beating had been confirmed as an effective method for disrupting cells prior to ethanol-based pigment extraction, the necessity of the subsequent maceration step was evaluated. To assess its impact, the extraction procedure was performed both with and without a maceration phase (1?hour at room temperature in darkness), and the resulting pigment yields were compared using a two-tailed t-test at a 95% confidence level. This assay was also conducted in triplicate.

Finally, a stability test was conducted to determine how long the ethanolic extract maintained the pigments in different storage temperatures: 4 and -$20^{\circ }$C. The extraction was done as before, and the extracts were distributed in black vials for their storage at the aforementioned temperatures. Pigment content was measured once a week for 4 weeks. This was done in triplicate.

### Membrane integrity comparation

This experiment was designed to assess whether differences in pigment extraction efficiency could be attributed to intrinsic differences in cell wall resistance to mechanical disruption, rather than to solvent effects. To address this question, flow cytometry analysis was performed to evaluate cell membrane integrity after bead beating. *S. almeriensis* cells cultured under heterotrophic and phototrophic conditions were disrupted as described above, except that no solvent was added and cells were suspended only in water. In parallel, *Chlorella vulgaris* cells from both metabolic regimes were analyzed to provide comparative insight across species.

After cell disruption, viability was assessed using fluorescein diacetate (FDA) and propidium iodide (PI), which exhibit complementary properties that facilitate the characterization of microalgae population viability. Specifically, the FDA distinguishes living cells (with valid esterase activity) from non-viable cells and debris. At the same time, PI enters dead cells and binds to their DNA, allowing the differentiation between non-viable cells (with compromised membrane permeability and DNA presence) and debris. The protocol described by Pozzobon et al. [43] was used to lead this assay. In brief, 200 $\upmu$L of 120 $\upmu$M FDA solution and 10 $\upmu$L of 1 g $\hbox {L}^{-1}$ propidium iodide (Sigma Chemicals) were added to 790 $\upmu$L of cell suspension at an optical density (750 nm) of 1.0±0.1. Afterward, cells were washed by centrifugation (15,000 rpm, $4^{\circ }$C, 5 min), and the supernatant was discarded. The pellet was resuspended in water before immediate analysis.

The dye signal was recovered using the yellow-green laser (561 nm, 610/20 nm detection) for propidium iodide and the blue laser (588 nm, 530/50 nm detection) for FDA. Heat-treated ($90^{\circ }$C, 10 min) cells were used as positive control (i.e., dead cells), and pristine cells were used as negative control (i.e., alive cells). Each of the tested conditions was duplicated.

Flow cytometry analyses were carried out using a BD Fortessa x20 (with BD FACS Diva software). Five parameters were recorded: forward scatter (or FSC, blue laser at 488 nm) as a proxy of cell size, side scatter (or SSC, blue laser at 488 nm, 488/10 nm detection) as a proxy of cell complexity, chlorophyll fluorescence (red laser at 620 nm, 780/60 nm detection), and propidium iodide fluorescence (yellow-green laser at 561 nm, 610/20 nm detection). At least 100 000 events (FSC above 5000) were acquired for each run.

### Pigment quantification

Pigments were quantified on an Ultima 3000 HPLC (Thermo Fisher Scientific) coupled with a UV detector. Separation was achieved on an Acclaim Polar Advantage II C18 column (4.6 x 150 mm, 3 $\upmu$m, 120 Å) from Thermo Fisher Scientific. The column temperature was maintained at $30^{\,\circ }$C. Pure methanol was the mobile phase. The flow rate was 0.5 mL $\hbox {min}^{-1}$, and the elution was set in isocratic mode. The injection volume was 5 $\upmu$L, and the total run analysis was 40 min. Compounds were identified by comparing their retention time and UV–Vis spectra with standard solutions. UV–Vis spectra were recorded from 200 nm to 700 nm. Absorbance was recorded at 400, 450, 500, and 650 nm. Pigment quantifications were led using the area of the peaks in external calibration for the most sensitive recorded wavelength. External calibration concentrations ranged from 0.25 to 5 mg $\hbox {L}^{-1}$. Pigment standards and methanol were purchased from Sigma-Aldrich. Standards had a purity greater than 97%. The five pigments of interest (chlorophyll a, b, lutein, violaxanthin, and zeaxanthin) were reported systematically for each sample. ‘N.A.’ was used whenever one could not be detected or quantified.

### Statistical analysis

Statistical significance was assessed using the ANOVA test. When the null hypothesis was rejected at a threshold of 0.05 (p < 0.05), the difference was considered significant. Then, the data were further analyzed using Tukey’s Honestly Significant Difference (HSD) test. The following results are presented as the average of the replicate (replication ranging from 2 to 5), while the error bars account for the standard deviation.

## Results and discussion

### Effect of biomass condition and storage

Lutein is a compound susceptible to oxidative degradation, accelerated by exposure to light and high temperatures. Under this scenario, most laboratory-level extractions seek to maintain the microalgal biomass in optimal conditions for lutein preservation while extraction processes are carried out. Traditionally, microalgal biomass is freeze-dried and stored at -$20^{\circ }$C to avoid changes in its composition while it is being processed for content analysis. However, this drying process constitutes the primary energy consumption of the operation and is notably time-intensive [54]. On the other hand, industrial-level operations can only afford to store biomass for a short period. This circumstance invites the exclusion of the drying step, enabling a direct transition from harvesting to extraction in a shorter period by simply cooling the wet biomass. In this context, it becomes crucial to evaluate the impact of extraction techniques on wet biomass, whether frozen at -$20^{\circ }$C or solely refrigerated at $4^{\circ }$C, which implies an energy reduction as well.

**Fig. 1:**
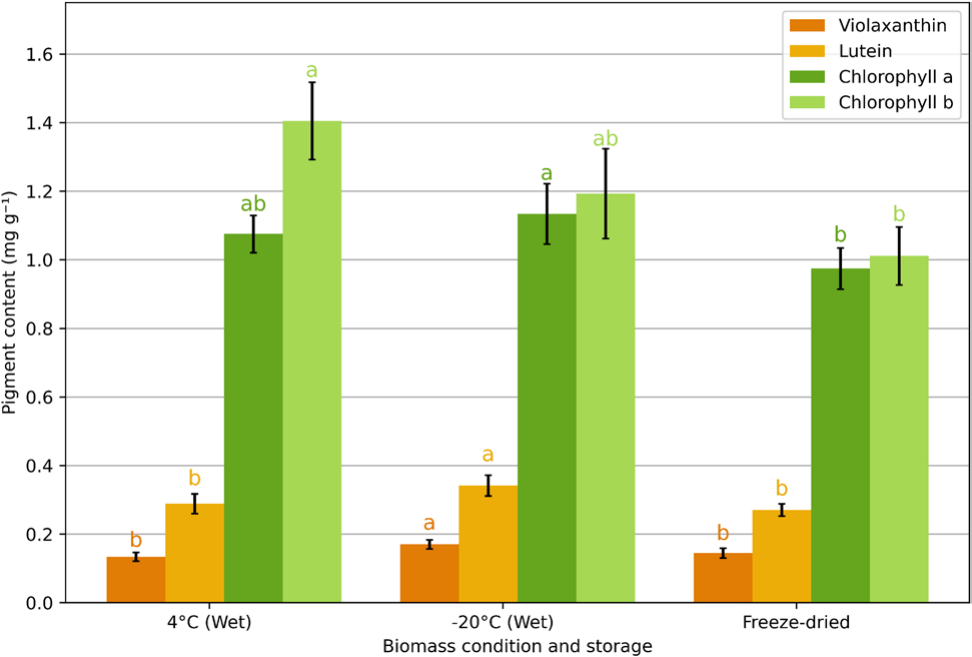
Pigment recovery from wet *S. almeriensis* biomass stored at $4^{\circ }$C and -$20^{\circ }$C compared to freeze-dried biomass. Methanol as solvent. Letters above bars indicate differences between pigment recovery, p < 0.05. n = 5

After removing the supernatant post-centrifugation, the samples utilized for wet biomass extraction exhibited a moisture content of 87±4%. Figure 1 shows the concentration of pigments of *S. almeriensis* from samples stored, for three days, at different temperatures and humidity percentages.

Samples extractions from wet samples stored at -$20^{\circ }$C resulted in the highest yields of violaxanthin (0.17±0.01 mg $\hbox {gDW}^{-1}$), lutein (0.34±0.03 mg $\hbox {gDW}^{-1}$) and chlorophyll a (1.13±0.08 mg $\hbox {gDW}^{-1}$), while chlorophyll b was recovered in higher amount from the wet samples preserved at $4^{\circ }$C (1.40±0.11 mg $\hbox {gDW}^{-1}$). It can be observed that freeze-drying the biomass has a negative effect on the recovery of chlorophyll a and b compared to samples preserved with humidity, either stored at $4^{\circ }$C or -$20^{\circ }$C, while storing the samples at -$20^{\circ }$C does not show a significant advantage compared to storage at $4^{\circ }$C for chlorophyll recovery. Regarding lutein and violaxanthin, storage of the wet biomass at -$20^{\circ }$C resulted in 1.18 and 1.27 times higher extraction than at $4^{\circ }$C, respectively. On the other hand, no differences were found between the amount of lutein and violaxanthin recovered from freeze-dried samples and wet biomass preserved at $4^{\circ }$C. These results are similar to those reported by Low et al. [35], where they obtained 1.39 times more lutein from wet biomass of *Scenedesmus sp.* compared to freeze-dried biomass. Similar findings are reflected in the results presented by Gong and Bassi [24]. Hence, wet extraction is recommended due to its higher yield and potential cost reduction by eliminating a drying step. The following tests in this work were performed with wet biomass stored at $4^{\circ }$C.

While these results only show the effectiveness of the extraction process on differently conditioned samples, further studies are needed to determine the full impact of freezing and drying the samples. Additionally, more studies are necessary to validate the stability of lutein on wet biomass for relatively long-term storage at $4^{\circ }$C. Low et al. [35] demonstrated that the wet biomass of *Scenedesmus* sp. ANI-KL 8D can be preserved for at least 21 days without loss of pigments, but this might be species-dependent and should be studied on a case-by-case basis.

Taken together, these results indicate that maintaining biomass hydration preserves solvent accessibility and reduces matrix compaction, which facilitates pigment diffusion during extraction. From a process perspective, this supports the feasibility of wet-biomass extraction as a means to reduce energy demand without compromising lutein recovery.

### Saponification effect

Naturally produced lutein can be found in two forms in plants and microalgae: esterified, with fatty acids at the ends of its 40-carbon chain, or in free form. In plants, it is commonly found esterified, and currently, the commercial process to obtain free lutein involves a process of saponification of marigold flower extracts [22]. Although there is no consensus on the form in which lutein is generally found in microalgae, some extraction protocols include a saponification process, either as a mechanism for cell disruption, to remove chlorophyll from the extract, or to release lutein from fatty acids if it is esterified [26]. However, the need to include this step is still under debate. As in many cases, this depends on the species and the conditions in which it was cultured, so it is necessary to define it for each process. Given that the predominant form of lutein in *S. almeriensis* is in free form [12], saponification in this instance aids primarily in disrupting the lipid membrane, thereby facilitating solvent penetration into the chloroplast.

#### Aqueous KOH

**Fig. 2:**
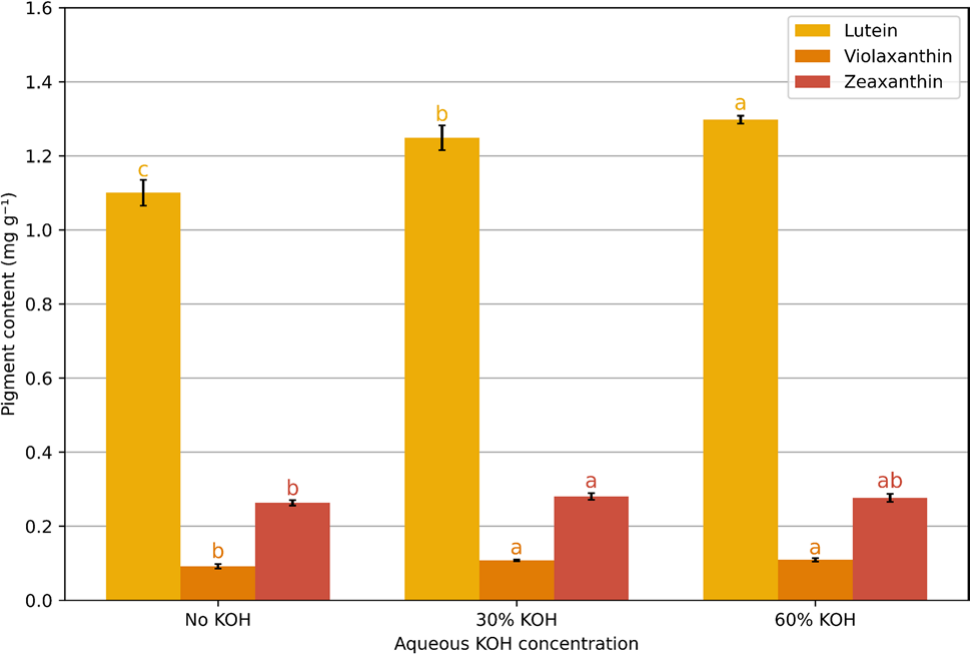
Carotenoids extracted with a saponification step after cell disruption of wet biomass using aqueous KOH (1 mL) and acetone as solvent (9 mL). Letters above bars indicate differences between pigment recovery, p < 0.05. n = 5

Figure 2 shows the amount of lutein, violaxanthin, and zeaxanthin recovered from wet biomass of *S. almeriensis* grown in heterotrophy and treated with aqueous KOH solution after cell disruption. It can be observed that the higher the concentration of KOH, the higher the pigments collected. Lutein recovered was 1.19 times higher using 60% KOH than without KOH, while it was 1.17 and 1.04 times higher for violaxanthin and zeaxanthin, respectively. It can also be observed that the recovered value of lutein is much higher in this extraction than that obtained in the previous methanol extraction, without saponification. This could be attributed more to the nature of the solvent (acetone) rather than to the saponification process itself, as even the extraction without KOH resulted in higher lutein content. The higher pigment results obtained by saponification are in agreement with results reported by Chan et al. [13], who achieved a 2-fold increase in the content of lutein from *S. obliquus* when employing a 2.5% KOH solution in contrast to omitting KOH. However, this same study reports that an increase in KOH concentration above 2.5% does not necessarily increase lutein recovery, which contrasts with the results reported in this work. In the same line, Gong et al. [23] exposes the need to use an alkaline solution to extract a higher content of lutein from wet biomass of *C. vulgaris*, however, in this case, the authors emphasize that it is necessary to obtain lutein in free form. Ceron et al. [12] also compared the concentration of KOH in the extraction of lutein from *S. almeriensis* and found that concentrations greater than 4% reduced lutein recovery. However, they did not compare it with lower concentrations.

#### Ethanolic KOH

**Fig. 3:**
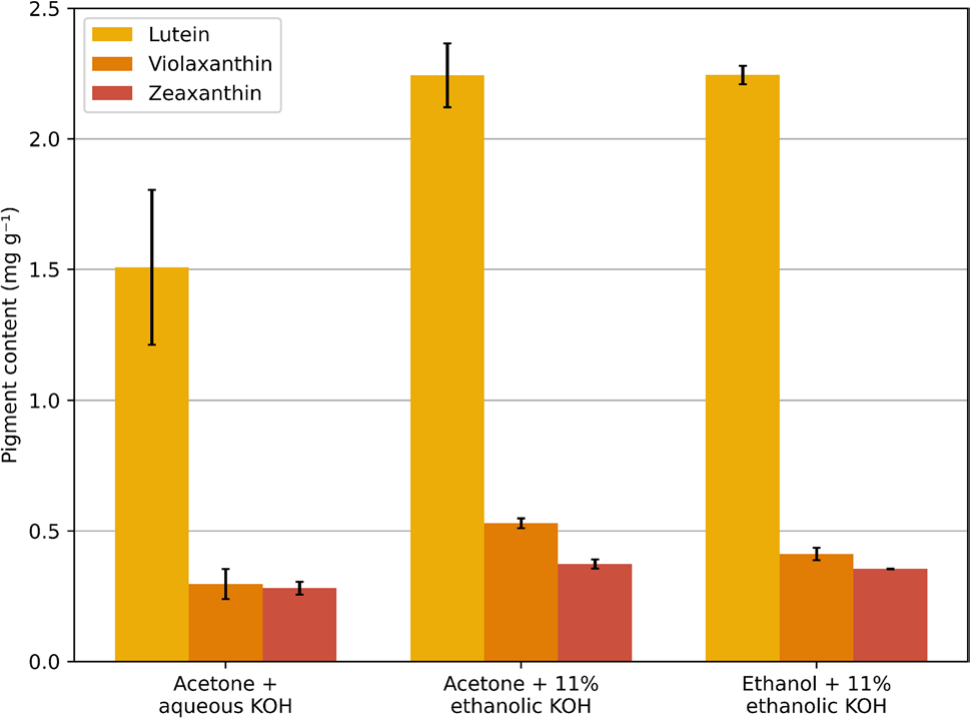
Carotenoids extracted using aqueous KOH during the saponification process compared to ethanolic KOH, either using acetone or ethanol as solvent. Mixture 9 mL of solvent and 1 mL of alkaline phase. Bead beating as homogenization method. Data presented as the average of the two replicates (n = 2), error bar covering the spread

Since lutein is not soluble in water, it has been suggested that using aqueous KOH presents the risk of precipitating part of the lutein in the saponification process [19]. For this reason, a comparison of carotenoid extraction using an aqueous KOH solution versus an ethanolic KOH solution was performed. An extraction using ethanol as a solvent was also performed to complete the comparison and rule out any interaction between acetone and ethanol. Figure 3 shows that the ethanolic KOH solution positively affects the recovery of carotenoids. Observed with the naked eye, using an aqueous solution results in the aqueous phase being divided from the organic phase by a thin opaque layer, which cannot be characterized but may contain saponified lipids and, possibly, part of the pigments.

On the other hand, using the ethanolic solution avoids the formation of phases. Moreover, lutein recovered using acetone plus ethanolic KOH was 32.80% higher than using aqueous KOH. Similar increases were observed for violaxanthin and zeaxanthin (43.83% and 24.94%, respectively). Since the extraction solvent was identical in all conditions, the observed improvement in lutein recovery with ethanolic KOH is attributed primarily to enhanced physicochemical solubilization and improved miscibility in the ethanolic alkaline phase rather than to the saponification reaction itself. These results are in agreement with D’Este et al. [19], who obtained three times more lutein with a solution of ethanolic KOH than aqueous KOH from *C. vulgaris* biomass. This is also explained by Low et al. [35], who argue that the mass transfer rate of lutein is limited by the insolubility of lutein in the aqueous KOH solution. Furthermore, no significant differences were observed when switching from acetone to ethanol as a solvent. Since ethanol is a solvent considered less toxic and is generally more accepted in the food industry, these results are promising for transitioning to a more environmentally sustainable process. In addition, it avoids the complexity of a purification step on a large scale. Indeed, two acetone molecules can condense through an aldol reaction to form diacetone alcohol. While slow and with low yield at room temperature, this reaction is catalyzed by alkali such as KOH [20], and may alter solvent performance.

These observations suggest that the enhanced recovery obtained with ethanolic KOH is primarily governed by improved physicochemical compatibility and mass transfer rather than by the saponification reaction itself, highlighting the limiting role of water in the extraction system.

### Solvent effect

Since the first results favored the use of wet biomass over dry biomass and the use of ethanolic KOH as the saponifying solution, it was necessary to go back in the solvent selection to rule out that any of these changes favored the extraction of carotenoids with solvents other than ethanol or acetone. Numerous studies have reported successful extraction processes using hexane, dichloromethane and other solvents that, due to their toxic nature, were discarded in this study, which aims to provide a more environmentally friendly and less toxic process for integration in food formulations.

Methanol, a solvent commonly used to extract polar pigments, such as xanthophylls, is considered by the U.S. Food and Drug Administration (FDA) to be Class 2, indicating that its use should be limited in pharmaceutical products due to its inherent toxicity. Ethanol, acetone and isopropanol are solvents also commonly used for pigment extraction and, according to the FDA, are solvents that pose no risk to human health at levels commonly accepted in pharmaceuticals (Class 3) [1]. According to this FDA report, no data assesses the Permitted Daily Exposure (PDE) of 2-MeTHF. However, according to Sicaire et al. [48], it is a solvent obtained from renewable sources by hydrogenation of hemicellulose from different feedstocks. In addition, it is reported to be biodegradable and easily recyclable.

**Fig. 4:**
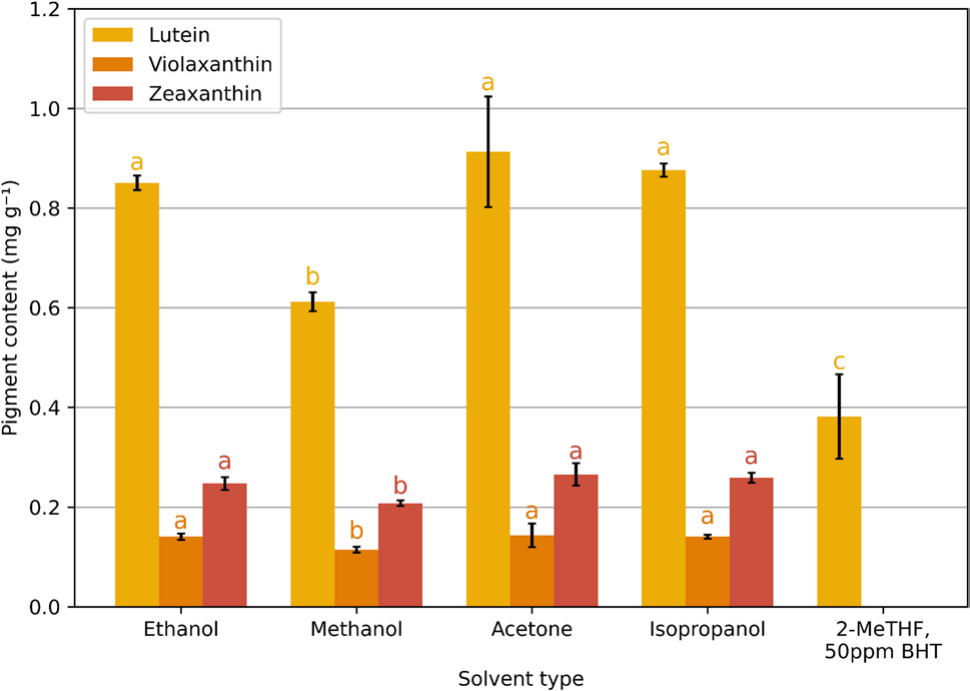
Carotenoids extracted from wet biomass using different solvents and 11% ethanolic KOH as saponification solution. Sonication as homogenization method. Letters above bars indicate differences between pigment recovery, p < 0.05. n = 5

As can be seen in Fig. 4, extraction with 2-MeTHF yielded the smallest amount of lutein (0.38±0.08 mg $\hbox {gDW}^{-1}$), while violaxanthin and zeaxanthin do not even appear in the HPLC chromatogram. Similar results were reported by Damergi et al. [17] on extracted lutein from *C. vulgaris*. Likewise, the values of lutein, violaxanthin and zeaxanthin extracted by methanol (0.61±0.01, 0.11±0.005, 0.$20\pm$0.005 mg $\hbox {gDW}^{-1}$) did not show advantageous results compared to ethanol (0.85±0.01, 0.14±0.006, 0.24±0.01 mg $\hbox {gDW}^{-1}$), acetone (0.91±0.11, 0.14±0.02, 0.26±0.02 mg $\hbox {gDW}^{-1}$) and isopropanol (0.87±0.01, 0.14±0.004, 0.25±0.009 mg $\hbox {gDW}^{-1}$). Although the extraction with ethanol was slightly lower than with acetone or isopropanol, this difference is not significant. In addition, the results showed less variation than with acetone.

Comparable results were achieved in a study conducted by Chen et al. [14], in which optimization of extraction conditions revealed that ethanol was better or at least as effective as other solvents studied in recovering lutein from *Chlorella* sp. Likewise, Ahmad et al. [2] obtained similar results in the extraction of xanthophylls using 90% acetone versus 90% ethanol from *C. luteoviridis* biomass. In contrast, Lee et al. [31] obtained 1.8 times more lutein from *Tetraselmis suecica* using methanol as solvent compared to ethanol or acetone. This difference in the solubility of lutein in various solvents may be caused by the difference in polarity of the solvents and the form in which the lutein is found. Gong et al. [23] explains that polar solvents extract lutein better from wet matrices, as in this study. In addition, they suggest using binary solvents, mixing one polar and one non-polar solvent to improve the solubility of lutein in its different forms (free and esterified). Moreover, Damergi et al. [17] suggests that the presence of water also influences the polarity of the extraction mixture, so it is necessary to differentiate between the use of wet and dry biomass in addition to the species in question. This shows that the choice of solvent is dependent on the species, and even more, on the conditions in which the microalgae were cultured and stored. Furthermore, Low et al. [35] highlights the limitation of using ethanol as a solvent for lutein extraction because it lacks selectiveness, with the chlorophyll in the final product. However, this is also true for acetone. Thus, further procedures are necessary to improve the purity of lutein extracts when ethanol is used as a solvent.

**Fig. 5:**
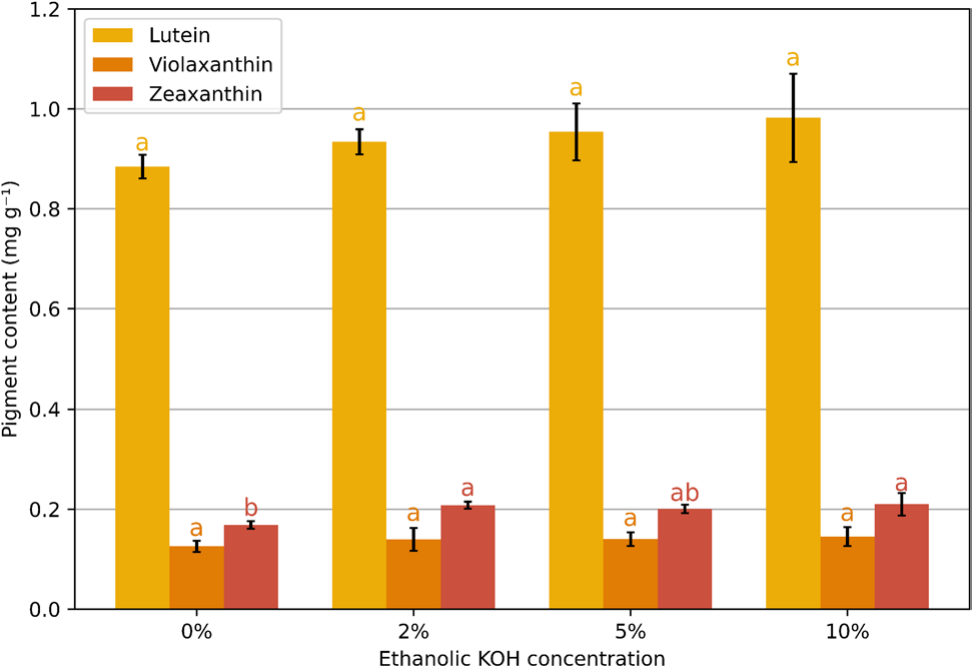
Carotenoids extracted from wet biomass using ethanol as solvent and different concentration of ethanolic KOH for saponification. Letters above bars indicate differences between pigment recovery, p < 0.05. n = 3

Once it was defined that ethanol is as good as acetone and isopropanol for carotenoid extraction, the next step was to evaluate the saponification’s effect at different concentrations of ethanolic KOH and using ethanol as the extraction solvent. As shown in Fig. 5, low concentrations of KOH (between 2 and 10%) did not favor lutein recovery compared to the lack of an alkaline solution. Despite the increase in recovered lutein being up to 9.91% higher when using 10% ethanolic KOH compared to not using saponification, the difference is not statistically significant, as opposed to the configuration with acetone, where this increase was significant. Therefore, under the tested conditions, saponification with ethanolic KOH does not provide a statistically significant benefit and can be omitted from the extraction protocol without compromising lutein yield.

To further confirm the robustness of the proposed protocol, its efficiency was assessed through additional tests. These included (i) verifying whether a single disruption-extraction cycle is sufficient to fully recover pigments, (ii) determining the necessity of the maceration step, and (iii) assessing the stability of extracted pigments over time under typical storage conditions.

Overall, the comparable performance of ethanol, acetone, and isopropanol indicates that solvent selection is dictated by functional compatibility with lutein and the wet biomass matrix rather than by absolute polarity alone, allowing process decisions to be guided by sustainability and regulatory considerations.

#### Multiple extraction

To determine whether a single disruption-extraction step was sufficient, sequential extractions were performed on the same biomass. Results (Table 1) confirmed that nearly all detectable pigments were recovered in the first cycle, with subsequent rounds yielding negligible additional amounts. Some of the reported values are slightly negative. These values result from analytical uncertainty around zero and indicate concentrations below the detection or quantification limit after the first extraction step. This supports the efficiency of the bead beating and ethanol extraction combination and suggests that one extraction round is sufficient for complete pigment recovery in *S. almeriensis*.

**Table 1:** Pigment content per extraction round

Extraction no.	Pigment (mg $\hbox {gDW}^{-1}$)			
	Violaxanthin	Lutein	Chlorophyll a	Chlorophyll b
**1**	0.17 ± 0.01	1.01 ± 0.03	2.84 ± 0.10	1.04 ± 0.03
**2**	0.00 ± 0.00	0.00 ± 0.01	0.00 ± 0.02	0.00 ± 0.01
**3**	0.00 ± 0.00	−0.01 ± 0.01	0.00 ± 0.02	−0.01 ± 0.01

Values are means of triplicate measurements (n = 3), error bars represent standard deviation

#### Maceration test

To assess whether maceration contributes to improved pigment extraction, the process was carried out with and without a one-hour maceration step. As shown in Table 2, no significant differences in pigment yield were observed between the two treatments. This result is consistent with the multiple extraction test, suggesting that pigment recovery is already maximized during the disruption phase. Eliminating the maceration step thus reduces processing time, increasing the method’s industrial feasibility.

**Table 2:** Pigment content extracted with and without maceration

Pigment	Not macerated (mg $\hbox {gDW}^{-1}$)	Macerated (mg $\hbox {gDW}^{-1}$)
Violaxanthin	0.59 ± 0.02^a^	0.61 ± 0.04^a^
Lutein	1.48 ± 0.22^a^	1.43 ± 0.23^a^
Chlorophyll a	7.92 ± 0.49^a^	7.81 ± 0.51^a^
Chlorophyll b	3.42 ± 0.08^a^	3.46 ± 0.15^a^

Values are means of triplicate measurements (n = 3), error bars represent standard deviation. Identical letters indicate no significant differences ($p > 0.05$)

#### Pigment stability

**Fig. 6:**
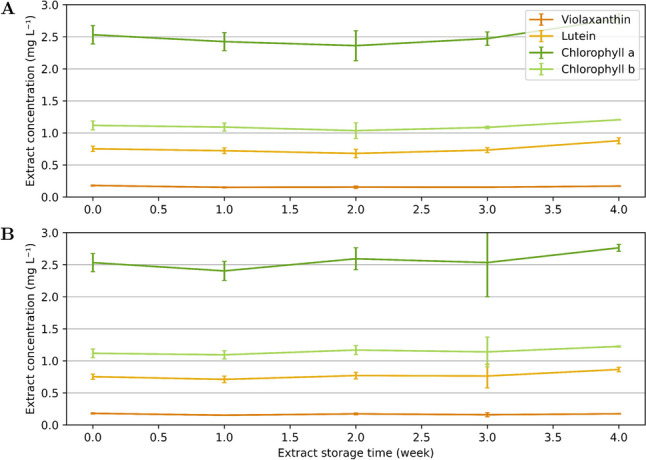
Pigment stability in ethanolic extracts stored at different temperatures over 4 weeks. (**A**) Stored at $4^{\circ }$C. (**B**) Stored at –$20^{\circ }$C. Values are means of triplicate measurements (n = 3), error bars represent standard deviation

Finally, the stability of pigments in ethanolic extracts was evaluated over four weeks of storage at –$20^{\circ }$C and $4^{\circ }$C. Figure 6 shows that pigment concentrations remained essentially unchanged at both temperatures. Indeed, observed variations are not statistically significant, and evaporation can be ruled out as vials’ masses were unchanged (weighed upon storage and upon withdrawal). Therefore, it can be concluded that the extracts are stable for at least a 4-week period at –$20^{\circ }$C and $4^{\circ }$C. This confirms that delayed analysis of stored samples is feasible without compromising pigment quantification, offering practical advantages in routine or large-scale processing. These findings are consistent with general principles of lipid preservation, as lipid-based compounds such as lutein are known to degrade in the presence of oxygen, light, and prooxidants due to autoxidation processes that generate peroxides and off-flavors [16]. In this study, extracts were stored in darkness and in containers with minimal headspace, which helped mitigate oxidative degradation. The combination of low temperature, reduced oxygen exposure (as samples were stored with minimal headspace containing air, but no neutral gas), and absence of light therefore contributed to the observed stability, validating the chosen storage strategy for practical applications.

#### Chromatographic validation of pigment extraction

**Fig. 7:**
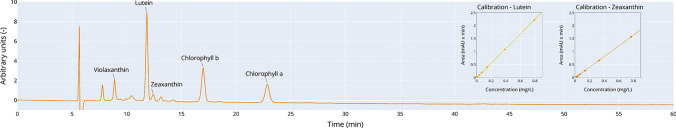
HPLC chromatogram of ethanol-extracted pigments from *Scenedesmus almeriensis* biomass. The major peak corresponds to free lutein, based on retention time compared to standards. No peaks corresponding to esterified lutein were observed at later retention times, confirming its predominance in the free form. Lutein and zeaxanthin calibration made on 7 and 6 points, repressively, with $\hbox {R}^2 > 0.99$

To further validate the nature of the extracted lutein, the chromatographic profile of the ethanol-extracted pigments was analyzed. As shown in Fig. 7, the main peak corresponding to free lutein was observed between 9 and 13 min, along with other free carotenoids, consistent with standard retention times reported in the literature [7, 30]. Importantly, no additional peaks were detected at later retention times, particularly in the 35 to 50-min range, where mono- and di-esterified carotenoids typically elute [7]. This absence of late-eluting signals confirms that lutein in *S. almeriensis*, under the tested cultivation and extraction conditions, is predominantly present in its free form. This observation aligns with previous reports for this species [12] and supports the conclusion that saponification is not required, as there are no esterified forms to hydrolyze for improved recovery.

### Streamlined protocol

The findings reported in this work can be streamlined into an easy-to-use protocol. Based on this work, the following procedure can be advised:

1. Solvent preparation: Prepare 2 M ethanolic KOH, if maximal recovery is intended (laboratory-scale quantification). Use pure ethanol otherwise.
2. (optional) Acquire culture concentration by the means of your choice: Dry mass, optical density to dry weight correlation,...
3. Harvest and dewater the cells: Centrifuge (16 000 g, 10 min, $4^{\circ }$C), discard supernatant.
4. Add solvent and disrupt: Add 1 mL of solvent per 1 mg of dry biomass. Bead beating at 6.5?m?$\hbox {s}^{-1}$ in two cycles of 30?seconds, with a 60-second pause between cycles, is recommended. Clean sand can be used as the crushing material.
5. Recover and filter the solvent with the extract: filter the extract (0.22 $\upmu$m).
6. Analyze: use the means of your choosing (HPLC, spectrophotometer and correlations,...).

Of course, the reader may adapt this protocol to suit its needs best. Among the points to be fine-tuned, the biomass-to-solvent ratio could be explored. This is especially true in the context of an industrial development where solvent minimization will be of interest for two reasons: minimizing the impact and cost of the process, as well as obtaining a concentrated extract. This remark complement the fact that solvent recycling is required to ensure economical viability at large scale [37]. Fortunately, ethanol recovery solutions are commercially available today. In addition, the fact that the proposed lutein recovery process takes place at room temperature is also a token of its scalability (during preliminary work, high temperature - $60^{\,\circ }\hbox {C}$ - was tested but did not yield any improvement). Finally, it is also important to note that among the different solvents encountered in the industry, ethanol exhibits similar properties (boiling point, latent heat of evaporation) compared to acetone and hexane, with much lower toxicity (Table 3).Also, ethanol can be deemed one of the most environmental-friendly solvent as evaluated by Capello et al. in their study ranking industrial solvent using a Environmental, Health and Safety (EHS) and Life-Cycle Assessment (LCA) criteria [11].

**Table 3:** Comparison of hexane, acetone, and ethanol

Solvent	Boiling point ($^{\circ }\hbox {C}$)	Latent heat of evaporation (kJ/mol)	TLV-TWA	TLV-STEL	Health effects
Hexane	68.8	28.9	50	–	Most
Acetone	56.1	30.3	250	500	Moderate
Ethanol	78.4	42.3	–	1000	Least

TLV–TWA - Threshold Limit Value–Time-Weighted Average, corresponding to the concentration to which it is believed that workers can be exposed 8-hour workday and a 40-h workweek. TLV–STEL - Threshold Limit Value–Short-Term Exposure Limit, corresponding to the concentration to which it is believed that nearly all workers can be exposed continuously for a short period of time. Source ACGIH. Health effects based on the datasheet from the New Jersey Department of Health

### Cell wall integrity comparison

Several studies have shown *S. almeriensis* as a strain with potential for industrial lutein production. Many of these studies have used different processes to extract lutein and illustrate this potential [12, 36, 38, 39, 51, 52]. However, the culture conditions in all these cases had been in phototrophic mode, which implies a possible change in cell structure when the cells are cultured in heterotrophy. These potential variations may explain discrepancies between these findings and those previously published. It is all the more important to lead this assessment, as, despite the lack of studies comparing the rigidity of the cell wall, there is a generalized idea (without literature support) that the cells of the genus *Scenedesmus* could be more recalcitrant than other genera of commercial interest, such as *Chlorella*, which can be considered the reference species for microalgal biotechnology. In this regard, Rashidi and Tindale [45] suggested that the amount of rhamnose in the cell wall is directly related to the rigidity of the cell wall. Additionally, Spain and Funk [49] reported that the concentration of rhamnose in the cell wall of *Scenedesmus* sp. was half that of *C. vulgaris* and Takeda [53] reports no rhamnose at all in eleven *Scenedesmus* species. This suggests that the cell wall of *S. almeriensis* may be less resistant to disruption than *C. vulgaris*.

For purposes of determining the cell wall resistance to the bead beating method described previously and without going further into the specific composition of each cell wall, comparisons were made between heterotrophically-grown and phototrophically-grown cells of *S. almeriensis* and *C. vulgaris* during the exponential growth phase. Disruption was carried out with the cells suspended only in water and not in a solvent to allow flow cytometer analysis and rule out the disruptive effect of the solvent. Cells were identified and gated using a 2D combination of their forward scatter (FSC) and side scatter (SSC) signals. As a safeguard, the obtained cluster was controlled for cluster unicity and absence of outliers, using the red channel fluorescence, i.e., chlorophyll fluorescence. Only then were PI and FDA signals used to identify pristine, broken but not stained, and stained broken cells.

**Fig. 8:**
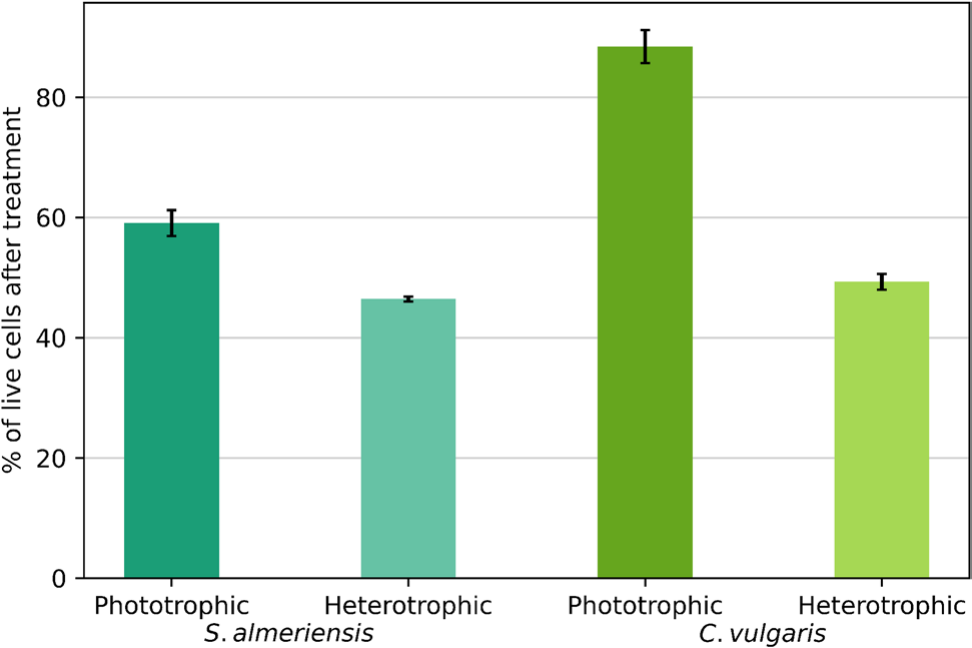
Percentage of viable phototrophic and heterotrophically-grown cells of *S. almeriensis* and *C. vulgaris* after bead beating disruption. Data presented as the average of the two replicates (n = 2), error bar covering the spread

Figure 8 shows the percentage of viable cells after disruption treatment. It can be seen that, for both *S. almeriensis* and *C. vulgaris*, a heterotrophically-grown culture has a negative effect on cell disruption resistance. In the case of *S. almeriensis*, cultures grown in phototrophy retained 59.05% viability, whereas only 46.38% of cells remained alive under heterotrophic conditions. For *C. vulgaris*, the disparity is even more pronounced, with 88.45% of phototrophic cells remaining viable compared to only 49.29% of heterotrophically-grown cells.

The comparison between the two species supports the argument above, confirming that *S. almeriensis* is less resistant than *C. vulgaris* when exposed to the same bead-beating treatment for cell disruption. To our knowledge, this is the first report that exposes the fragility of different species in two different culture modes to the same cell disruption treatment.

Although the results of this test determine the fragility of the cell wall of these species, resistance is not necessarily linked to composition, as it is known that other factors, such as cell size and shape, can determine the degree of disruption [3].

Most studies that analyze the relationship between cell wall composition and the effectiveness of different cell disruption methods perform this analysis on a single species and in a single culture condition [3, 28]. On the other hand, studies that characterize the cell wall rarely correlate it with analyses of resistance to disruption [5, 10]. Moreover, although it is recognized that changes in the structure and composition of the cell wall of microalgae occur at different times of culture (i.e., exponential or stationary phase) [10], there are no studies that make this comparison between cultures grown in different metabolic regimes or their impact on disruption resilience.

Further research is needed to elucidate the structural and biochemical alterations contributing to cell wall toughness under different metabolic regimes. Nevertheless, these findings provide valuable information for microalgal biomass downstream processing.

## Conclusions

Microalgae culture is a promising and attractive source of lutein at the industrial level; however, the high costs of the process and the complexity of scaling it up to the industrial level present a barrier that needs to be addressed to make it commercially available. In addition, it is becoming increasingly necessary to address the challenge of making the process less toxic for product formulation and the environment.

The results of this study highlight that, at laboratory scale, freeze-drying the *S. almeriensis* biomass cultured heterotrophically does not lead to increased lutein recovery, suggesting that alternative storage methods, such as refrigeration at $4^{\circ }$C, can be viable options while maintaining extraction efficiency. Moreover, ethanolic KOH solution demonstrates superior efficacy for lutein extraction compared to aqueous solution, with saponification offering marginal benefits. Among the solvents examined, ethanol emerges as a promising alternative, outperforming methanol or 2-methyltetrahydrofuran and performing comparably to acetone or isopropanol. These findings demonstrate the potential for reducing the environmental footprint and improving the efficiency of lutein extraction processes. However, while the results are promising at laboratory scale, further validation is necessary under industrial conditions, where factors such as energy input, solvent recovery, and scalability of mechanical disruption could significantly influence performance. Moreover, economic analyses are needed to assess the cost-effectiveness of the optimized protocol and confirm its industrial viability. Observations regarding the lower resistance of *S. almeriensis* cells cultivated under heterotrophic conditions to mechanical disruption may also inform more efficient downstream processing strategies.

## Data Availability

Data are available upon request.
